# Storage-induced mechanical changes of porcine lenses assessed with optical coherence elastography and inverse finite element modeling

**DOI:** 10.3389/fbioe.2024.1398659

**Published:** 2024-06-13

**Authors:** Vahoura Tahsini, Iulen Cabeza Gil, Sabine Kling

**Affiliations:** ^1^ ARTORG Center for Biomedical Engineering Research, University of Bern, Bern, Switzerland; ^2^ Aragón Institute of Engineering Research (i3A), University of Zaragoza, Zaragoza, Spain

**Keywords:** optical coherence elastography, inverse finite element analysis, crystalline lens, hyperelastic material, preservation condition

## Abstract

**Introduction:**

In an effort of gaining a better understanding of the lens mechanics, *ex vivo* lenses samples are often used. Yet, *ex vivo* tissue might undergo important postmortem changes depending on the unavoidable preservation method employed. The purpose of this study was to assess how various storage conditions and the removal of the lens capsule affect the mechanical properties of *ex vivo* porcine lens samples.

**Methods:**

A total of 81 freshly enucleated porcine eyes were obtained and divided into six groups and preserved differently. In the first three groups, the lens within the intact eye was preserved for 24 h by: (i) freezing at −80°C (n = 12), (ii) freezing at −20°C (n = 12), and (iii) refrigeration at +8°C (n = 12). In the remaining groups, the lenses were immediately extracted and treated as follows: (iv) kept intact, no storage (n = 12), (v) decapsulated, no storage (n = 21), and (vi) immersed in Minimum Essential Medium (MEM) at +8°C (n = 12) for 24 h. Frozen lenses were thawed at room temperature. Each lens was compressed between two glass lamella and subjected, first to a period of relaxation during which the compression force was recorded and second to an oscillating micro-compression while the deformation was recorded with a total of 256 subsequent B-scans via optical coherence tomography. The corresponding axial strain was retrieved via phase-sensitive image processing and subsequently used as input for an inverse finite element analysis (iFEA) to retrieve the visco-hyperelastic material properties of the lenses.

**Results:**

After freezing at temperatures of −80°C and −20°C, the cortical strains increased by 14% (*p* = 0.01) and 34% (*p* < 0.001), and the nuclear strains decreased by 17% (*p* = 0.014) and 36% (*p* < 0.001), compared to the lenses tested immediately after postmortem, respectively. According to iFEA, this resulted from an increased ratio of the nuclear: cortical E-modulus (4.06 and 7.06) in −80°C and −20°C frozen lenses compared to fresh lenses (3.3). Decapsulation had the largest effect on the material constant C_10_, showing an increase both in the nucleus and cortex. Preservation of the intact eye in the refrigerator induced the least mechanical alterations in the lens, compared to the intact fresh condition.

**Discussion:**

Combining iFEA with optical coherence elastography allowed us to identify important changes in the lens mechanics induced after different preserving *ex vivo* methods.

## 1 Introduction

The most important refractive components of the eye are the cornea and the crystalline lens. The latter provides approximately one-third of the refractive power and has the unique characteristic to change its shape and adjust the focal length of the eye to different distances, which is referred to as accommodation. During accommodation, the diameter of the lens changes by contracting the ciliary muscles and relaxing the tension on the zonular fibers. This goes along with a large mechanical deformation that heavily depends on the inherent material properties of the lens. In terms of its mechanical properties, the lens capsule has previously been reported to play an important role in the lenticular deformation behavior (Reilly and Cleaver, 2017), particularly with respect to the lens’ viscoelastic properties (Mekonnen et al., 2023). In an effort to get a better understanding of accommodation, the deformation behavior of the crystalline lens during accommodation has mostly been investigated by means of macroscopic changes in the lens thickness, diameter, and curvature assessed with diverse imaging techniques, including ultrasound (Yoon et al., 2012; Zhang et al., 2018) and magnetic resonance (Sheppard et al., 2011), but predominantly via optical imaging techniques (Purkinje, Scheimpflug, Optical Coherence Tomography). Other studies have examined the mechanical deformation behavior of the *ex vivo* crystalline lens under controlled loading conditions, such as during spinning (Wilde et al., 2012; Reilly et al., 2016) or while mounted in a lens stretcher (Marussich et al., 2015; Heilman et al., 2018; Martinez-Enriquez et al., 2020). Due to their *ex vivo* nature, most of these mechanical characterization studies on crystalline lenses have been conducted in human donor tissues. It is critical that the *ex vivo* lens is adequately preserved after harvesting to prevent degradation and maintain its physiological characteristics. While low temperature helps maintain cell viability for long periods, its effect on the tissue’s mechanical characteristics has hardly been addressed. The difference in mechanical properties due to storage of the intact eye in the refrigerator has previously been assessed with spinning tests (Fisher, 1971), and of extracted lenses in saline solution in the refrigerator with indentation tests (Czygan and Hartung, 1996), where no relevant differences were reported. Freezing is another common preservation technique, but it had previously been demonstrated to affect the viscoelastic behavior of the lens (Weeber et al., 2005). Another study has reported a minor softening effect of the lens capsule in response to freezing (Krag and Andreassen, 1998). Yet, it remains unclear whether cortical and nuclear regions are similarly affected by storage and which is the most suitable preservation method in terms of not altering the mechanical properties. Another common preservation for the crystalline lens (Jones et al., 2005) is minimum essential medium (MEM), which provides cells with essential components necessary for survival, growth, and proliferation. To our knowledge, the effects of preservation in MEM on the lens mechanics have not been studied before.

So far, all of these studies have in common that internal lenticular deformations could only be indirectly retrieved by inverse modeling (Weeber et al., 2007; Weeber and Van Der Heijde, 2008). In this context, inverse Finite Element Analysis (iFEA) is a valuable technique to identify mechanical parameters either from non-invasive geometrical measurements, or in materials with complex mechanical properties, and with patient-specific geometries (Lanchares et al., 2012). Applied to the lens, iFEA previously allowed to quantify the change in material properties underlying presbyopia (Lanchares et al., 2012) by considering the accommodation amplitude at different ages. However, due to the highly nonlinear properties of the lens, material model selection and material parameter identification remains challenging.

Recently, Brillouin microscopy has provided the first highly-resolved stiffness maps (Besner et al., 2016) of the aging human lens measured *in vivo*. Yet Brillouin scattering depends on the refractive index and the tissue’s hydration state, which could be severely affected by the gradient distribution within the lens. This would explain the discrepancy of the retrieved stiffness distribution with Brillouin compared to those expected from earlier studies. Optical coherence elastography (OCE) is an emerging technique for characterizing tissue mechanics (Kling, 2020) with high spatial resolution. In the past, air-puff based OCE has shown promising results to identify differences in lens stiffness both, at different intraocular pressures (Wu et al., 2018) and with age (Li et al., 2019; Zhang et al., 2022). Previously, we applied phase-sensitive quasi-static OCE in combination with iFEA to quantify the visco-hyperelastic mechanical properties of the porcine lens under an oscillating compression. Due to the higher resolution compared to earlier macroscopic relaxation measurements (Sharma et al., 2011; Alzoubi et al., 2024), we were able to quantify nuclear and cortical regions separately, and to find out that viscoelasticity was only present in the nucleus.

In the current study, we apply the same technique to study the effect of the lens capsule and different preservation conditions on the mechanical properties of the lens. Importantly, we perform a mechanical comparison of different regions within the lens. This investigation is particularly relevant, as many *ex vivo* studies on accommodation have been performed in donor eyes that have been preserved for different durations before the measurement was conducted. As such, the current study will enable a better comparison of earlier works using different preservation techniques.

## 2 Materials and methods

Optical Coherence Elastography (OCE) and inverse Finite Element Analysis (iFEA) were combined in the current study to quantify the axial strain of the crystalline lens during an oscillating compression and inversely retrieve its biomechanical properties.

### 2.1 Samples and preservation conditions

A total of 81 porcine lenses were obtained from the local slaughterhouse and prepared as follows, see also Table 1: Crystalline lenses of eyes in group 1 were excised in the freshly enucleated eye and tested immediately. Lenses of group 2 were excised in the freshly enucleated eye, subsequently decapsulated and tested immediately. Eyes in group 3 were stored intact at +8°C for 24 h before the crystalline lenses were excised and tested. Crystalline lenses of eyes in group 4 were excised in the fresh eyes and subsequently stored in Minimum Essential Medium (MEM) for 24 h at 8°C before the measurement. Eyes in group 5 were stored intact at −20°C for approx. 24 h before the eyes were allowed to thaw at room temperature. Subsequently the crystalline lenses were excised and tested. Eyes in group 6 were stored intact at −80°C for approx. 24 h before the eyes were allowed to thaw at room temperature, similar as in previous studies (Weeber et al., 2007).

**TABLE 1:** Summary of the experimental conditions assessed in this study.

Group	Name	Sample size	Storage	Preservation time (h)	Lens extraction	Decapsulation
1	fresh	12	tested immediately	0	immediately	no
2	de-cap	21	tested immediately	0	immediately	yes
3	fridge8	12	+8°C intact	24	after 24 h	no
4	MEM8	12	+8°C in MEM	24	immediately	no
5	frozen20	12	−20°C intact	24	after 24 h	no
6	frozen80	12	−80°C intact	24	after 24 h	no

**FIGURE 1:**
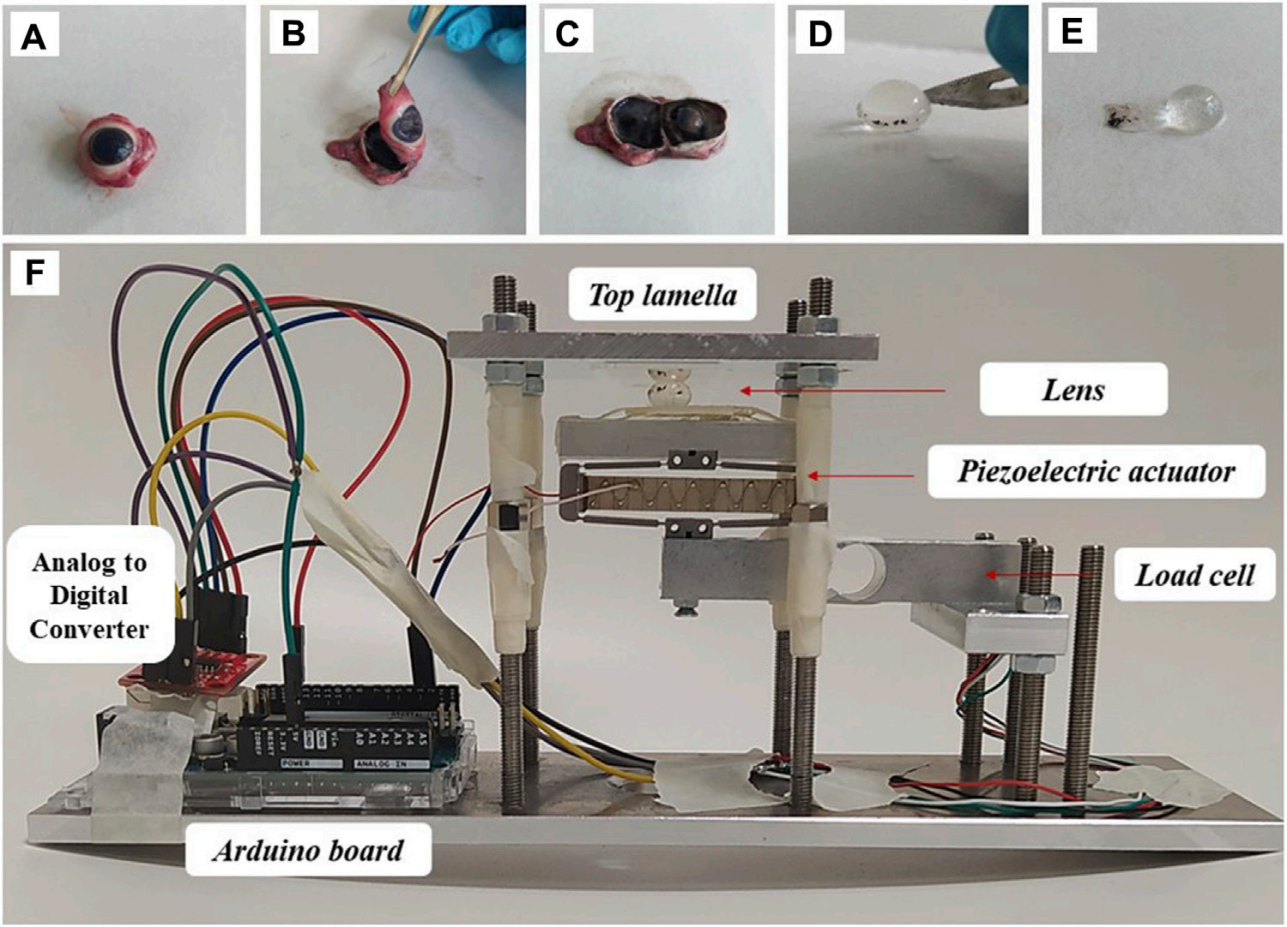
Sample preparation and experimental set-up. **(A–C)** Individual steps of lens separation, **(D,E)** lens decapsulation, **(F)** mounting in the compression setup.

For crystalline lens extraction (Figures 1A–E), a scalpel was used to place an incision at the scleral equator. Then the anterior part of the eyeball was separated by micro-dissection scissors. After removing the aqueous humor, the lens was dissected from the ciliary muscle. For group 2 lenses, the capsule was dissected by carefully cutting along the lens equator before peeling the capsule off with a set of tweezers.

### 2.2 Geometrical assessment

**FIGURE 2:**
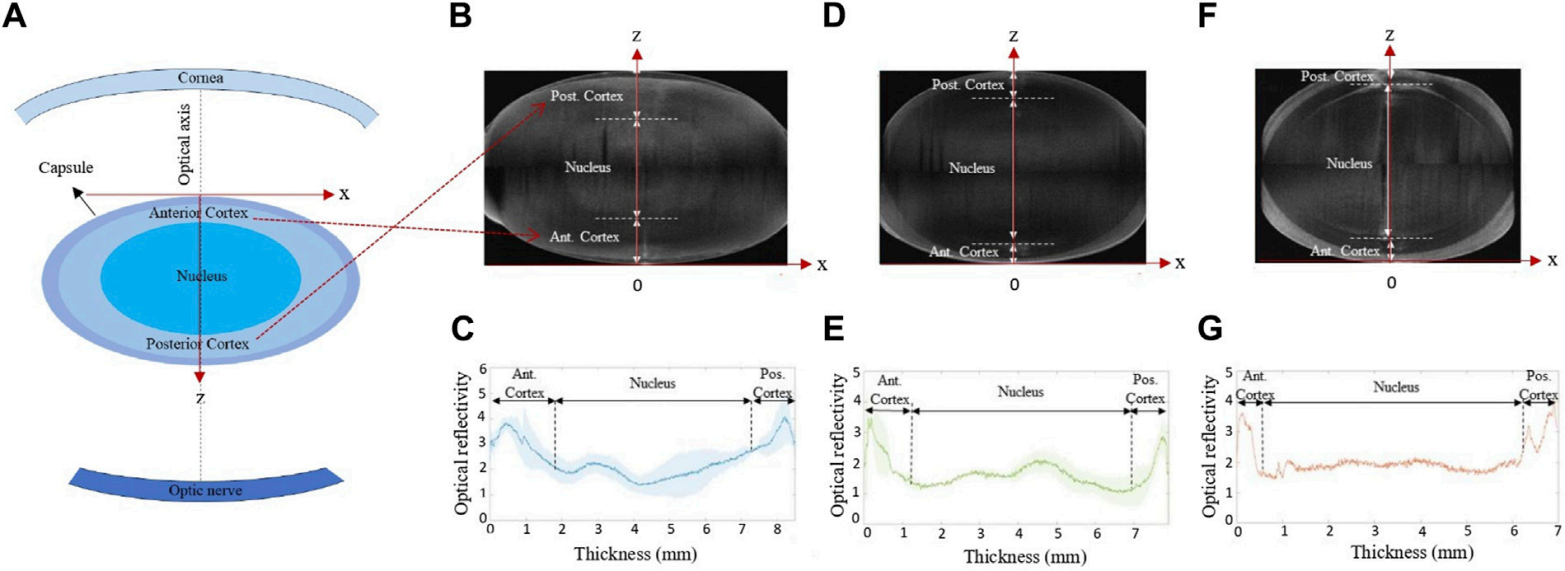
**(A)** Sketch of the lens position within eye. **(B,D,F)** Representative cross-sectional OCT images and **(C,E,G)** mean intensity profile of the lenses before compression, respectively, averaged for the conditions **(B,C)** frozen at −20°C, −80°C and MEM, **(D,E)** fresh and refrigerator, and **(F,G)** after decapsulation.

Before the actual mechanical characterization was conducted, the intact lens geometry was recorded. All geometric assessments and mechanical measurements were conducted at room temperature. For this purpose, a commercial optical coherence tomography device (Anterion, Heidelberg Engineering, Germany) was used, with an axial and lateral resolution of 9.5 µm (in air) and 35 μm, respectively. Two structural OCT scans were conducted: one with the anterior side of the lens facing towards the OCT, and one with the posterior side facing towards the OCT. The two scans were then merged into a single scan with extended depth. Subsequently, the corresponding reflectivity profiles were retrieved and used to identify the lens nucleus and cortical regions, see Figure 2.

### 2.3 Mechanical stimulation

Two separate measurements were performed to fully determine the mechanical properties of the lens:

(i) Stress relaxation. For this purpose, the distance between the two glass lamellas was reduced to 5.4 mm for lenses with capsule, and to 4.4 mm for the decapsulated group, to induce an engineering pre-strain of the lens of 33%. The distance between the two lamella and thus of the compressed lens thickness was measured by OCT (Figure 1F). A load cell weight sensor (HX711, max. load 1 kg) positioned under the piezo electric actuator was used to record the force relaxation for a duration of 2 min (short) and 30 min (long). Force was measured in gram-force (1 gf = 9.8 mN). The relatively high initial pre-strain was necessary to achieve (a) a reliable force measurement and (b) a compression over a larger proportion of the lens.
(ii) Oscillating compression. For this purpose, the pre-compressed lens was subjected to a sinusoidal compression/relaxation cycle by means of a piezoelectric actuator (APF705, Thorlabs, USA), which displaced the bottom lamella with a stroke length of −32–36 µm. The latter was quantified previously (Cabeza-Gil et al., 2023). Simultaneously to the oscillating displacement, the dynamic strain distribution within the lens was quantified.

### 2.4 Optical coherence elastography

For dynamic strain assessment during the oscillating compression, a total of 256 subsequent OCT B-scans were acquired at the same location with a frame rate of 33 Hz, resulting in an overall measurement duration of 7.6 s. The displacement that occurred between two subsequent B-scans was determined by phase-sensitive processing of the complex-valued OCT signal as described recently (Zaitsev et al., 2016; Kling, 2020). Briefly, the axial displacement is calculated from Eq. 1:

$$∆z=\frac{\lambda .∠R}{4\pi .n\left(z\right)},$$ (1)

where λ = 1200 nm is the central wavelength of the OCT, n(z) is the gradient refractive index of the porcine lens and ∠R is the angle of the complex cross-correlation which is retrieved from Eq. 2:

$$R=\sum_{j=-w_{z}}^{w_{z}}\sum_{k=-w_{x}}^{w_{x}}B_{S}\left(z+j,x+k\right)\;\cdot \;B_{S+1}^{*}\left(z+j,x+k\right),$$ (2)

where B represents an OCT B-scan, B* its complex conjugate, and s = {1, 256} is the number of B-scans. Phase-processing windows with a size of *w*_z_ = 10 and *w*_x_ = 1 pixels were applied. Strain was approximated as the axial gradient and computed by applying a second cross-correlation with the by one pixel axially shifted first complex cross-correlation, according to Eq. 3:

$$\varepsilon_{z}=\frac{∆z∙n\left(z\right)}{asu}=\frac{\;\lambda \cdot ∠\sum_{l={-v}_{z}}^{v_{z}}\sum_{m={-v}_{x}}^{v_{x}}\left(R_{s}\left(z+l,x+m\right)\cdot R_{s}^{*}\left(z+1+l,x+m\right)\right)}{4\pi \;\cdot asu},$$ (3)

being asu = 9.5 μm the axial sampling unit (in air), which means the axial dimension of a pixel. *v*_z_ = 15 and *v*_x_ = 1 pixels were the applied strain processing windows. Note that in contrast to displacement, axial strain measured with OCE is independent of the refractive index. Also, due to the compression between two flat glass lamella, no optical distortions due to the lens geometry are expected.

Cross-sectional axial strain was recorded. In order to improve signal quality, strains were averaged across a lateral zone of 2.3 mm. Subsequently, the average strain within three distinct regions(anterior cortex, posterior cortex and the nucleus) was computed for comparison among the groups. The corresponding regions were identified from the structural OCT image (see Figure 2). For validation, the average OCE strain across the full lens thickness was computed and a homogenous refractive index (Regal et al., 2019) of 1.49 was assumed to determine the corresponding lamella distance in order to compare the OCE-measured compression with the macroscopic compression applied by the piezoelectric actuator.

### 2.5 Finite element modeling

In order to retrieve the mechanical properties of the lenses tested experimentally under different conditions, inverse finite element analysis (iFEA) was performed in Abaqus (version 6.14, Dassault Systèmes). For this purpose, a 2D axisymmetric finite element (FE) model was developed to simulate the experiment. The glass lamella were modeled as rigid bodies, whilst the lens shapes were obtained from structural OCT scans of the lens without any zonular anchoring taken before any mechanical tests were performed. A representative geometry was determined for every condition.

**FIGURE 3:**
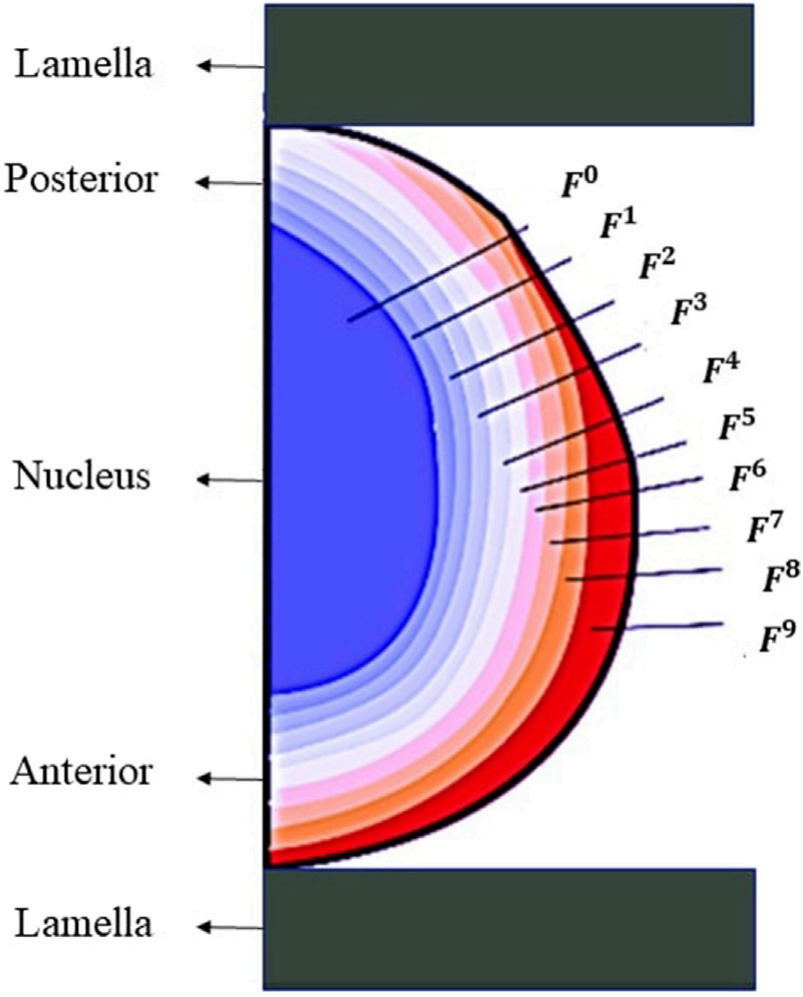
Geometry used for finite element modeling.

The lens geometry was composed of the nucleus, the cortex and the capsule. To simulate the previously reported stiffness (Weeber et al., 2007) gradient within the lens and to account for the anatomical layered structure of the lens fiber cells (Kuszak, 1995; Taylor et al., 1996), the anterior part was divided into nine layers of cortex whilst the posterior was divided into 6, see Figure 3. This division was made to describe the difference in stiffness observed in the experimental tests between the anterior and posterior cortex of the lens. The stiffness of every layer of the cortex was defined by Eq. 4:

$${Cortex\left(n\right)=N}_{stiffness}\cdot \;{F^{n}\cdot \;C}_{10-N}^{\infty },$$ (4)

where n in the exponent is the number of the respective cortical layer, being n = 1 the cortical layer closest to the nucleus. F is a form factor to differentiate the stiffness across the cortical layers. N is used to increase the relative stiffness of the nucleus with the cortex, and $C_{10-\left(N\right)}^{\infty }$ is the long-term Neo-Hookean modulus of the nucleus. Because the thickness of the posterior cortex is thinner than the anterior one, it contains only six layers. The anterior cortex contains all nine layers. To better describe the elasticity of the anterior and posterior cortex in the results section, the $C_{10-\left(n\right)}^{\infty }$ modulus was averaged as the stiffness of the layers that compose the anterior or posterior cortex, respectively, giving an average ${\bar{C}}_{10-ANT}$ and ${\bar{C}}_{10-POST}$ .

Initially, the top lamella axially compressed the lens to a thickness of ${LT}_{test}={LT}_{0}-∆$ . This step was performed to assure the lens stability in the setup. The undeformed thickness LT _0_ of the decapsulated lenses was notably lower (6.6 mm) compared to all lenses with an intact capsule (8.06 mm). Therefore, ${LT}_{test}=4.40\;mm$ was used for decapsulated lenses and ${LT}_{test}=5.40\;mm$ for the other conditions, such that a similar compression of 33% was achieved. After the initial compression, an experimental sinusoidal micro-displacement was performed by the bottom lamella. The strains were calculated with the compressed lens configuration as a reference.

Large strains and nonlinearity were considered in this dynamic simulation. The axisymmetric conditions were imposed in the model about the *y*-axis. A “hard contact” behavior, strictly prohibiting penetration between the lamella and lens surfaces, was applied to maximize the realism of the simulation. No friction was applied.

The lens can be described as a visco-hyperelastic material consisting of the nucleus, cortex and capsule. The importance of accounting for the lens capsule has recently been demonstrated in a simulation study (Reilly and Cleaver, 2017) under a similar compressive loading. Therefore, following the approach of our recent study (Cabeza-Gil et al., 2023), the lens capsule was modeled with a homogeneous thickness of 60 μm and a Neo-Hookean coefficient of 0.166 MPa (equivalent to a Young’s modulus of 1 MPa). Due to the substantial strain induced during the pre-compression step, the lens nucleus was characterized by an incompressible visco-hyperelastic Neo-Hookean material model with a strain energy density function of Eq. 5:

$$\psi \left(C,t\right)=C_{10-N}^{R}\left(t\right)\;\left(I_{1}-3\right),$$ (5)

being $C_{10-N}^{R}\left(t\right)$ the hyperelastic Neo-Hookean coefficient and $I_{1}$ the first invariant of the right Cauchy-Green deformation tensor. The time dependence of $C_{10-N}^{\infty }\left(t\right)$ is defined by a one term (*N* = 1) Prony series according to Eqs 6 and 7:

$$C_{10-N}^{R}\left(t\right)=C_{10}^{0}\;\left(1-\;\sum_{i=1}^{N}g_{i}\left(1-\;e^{-\frac{t}{\tau i}}\right)\right),$$ (6)

$${C_{10}^{\infty }=C}_{10}^{0}\left(1-\;\sum_{i=1}^{N}g_{i}\right),$$ (7)

where $C_{10}^{0}$ is the instantaneous modulus and $C_{10}^{\infty }$ the long-term modulus. The Prony series parameters are defined by the pre-exponential factor g_1_ and the relaxation time τ_1_.

For consistency with linear elasticity in small deformations and for comparison across studies, the incompressible Neo-Hookean model can be converted to a linear elastic model according to Eqs 8 and 9 with the following relationship:

$$µ=2C_{10\;},$$ (8)

E=3µ, (9)

which µ is the first Lamé parameter, and E, the Young’s modulus.

The lens cortex was modeled with a hyperelastic Neo-Hookean material model, without effect of any viscous behavior as we did not observe this previously. (Cabeza-Gil et al., 2023).

#### 2.5.1 Inverse finite element analysis

A response surface was generated to inversely retrieve the mechanical properties for all the lenses under investigation. A response surface was generated with a full factorial design for every lens preserving condition, involving the thickness of the lens, the Prony series terms of the lens nucleus (τ_1_), and the stiffness ratio $\frac{C_{10-N}^{\infty }}{{\bar{C}}_{10-ANT}},\frac{C_{10-N}^{\infty }}{{\bar{C}}_{10-POST}}.$ The Prony term, *g*_*1*_ (*−*), was set to 0.25 for all preserving conditions in order to reduce overfitting and consequently allow a better comparison across all samples (Cabeza-Gil et al., 2023).

Three to four levels of thickness, seven levels of τ_1_, and 10 levels of the parameter F and N (100 combinations) were varied for every preserving lens condition to do the inverse fitting. Each range parameter was chosen based on screening analysis trying to cover the behavior of all samples of one condition. There was no geometrical difference between short and long-term relaxation measurements, therefore the same lens geometries and response surfaces were used to retrieve the mechanical properties. Table 2 summarizes the full factorial design used to characterize each preserving lens condition. This resulted in approximately 2000 simulations for every lens conditions.

**TABLE 2:** Parameters and their ranges used for the response surface.

	Thickness	Tau	F	N
fresh, fridge8	[7.1, 7.4, 7.55, 7.8]	[0.25, 0.5, 1, 1.5, 3, 5, 7.5]	Ranging from 1.0 to 3.0 in increments of 0.25	Ranging from 0.65 to 1.05 in increments of 0.05
de-cap	[5.7, 6.3, 6.8, 7.3]	[0.25, 0.5, 1, 1.5, 3, 5, 7.5]	Ranging from 1.0 to 4.0 in increments of 0.4	[0.85 in 0.03 increments up to 1.03] Ranging from 0.85 to 1.03 in increments of 0.03
MEM8	[8.1, 8.4 8.8]	[0.25, 0.5, 1, 1.5, 3, 5, 7.5]	Ranging from 1.0 to 3.0 in increments of 0.25	Ranging from 0.65 to 1.05 in increments of 0.05
frozen20, frozen80	[8.3, 8.8, 9.2]	[0.25, 0.5, 1, 1.5, 3, 5, 7.5]	Ranging from 2.0 to 6.0 in increments of 0.25	Ranging from 0.85 to 1.15 in increments of 0.05

After generating the response surface with the full factorial design, an optimization process was carried out in Minitab to inversely retrieve the mechanical properties of each lens ( $\frac{C_{10-N}^{\infty }}{{\bar{C}}_{10-ANT}}$ ; $\frac{C_{10-N}^{\infty }}{{\bar{C}}_{10-POST}}$ , τ_1_, F, N_stiffness_) from the experimental values. The optimization was performed by minimizing an error metric (SSE) see eq. 10 defined as the relative average sum of the maximum and minimum strains in the anterior cortex, posterior cortex, and nucleus, along with the viscoelastic delay. All outputs had the same weight.

$$SSE=\sum_{i=1}^{n}{\left(\alpha_{i}-\kappa \left(x_{i}\right)\right)}^{2}+{\left(\beta_{i}-\lambda \left(x_{i}\right)\right)}^{2}+{\left(\gamma_{i}-\mu \left(x_{i}\right)\right)}^{2}+{\left(\delta_{i}-\nu \left(x_{i}\right)\right)}^{2},$$ (10)

where $\alpha_{i},\beta_{i},\gamma_{i}$ are the measured strain amplitudes at the $i^{th}$ data point of the anterior cortex, posterior cortex, and nucleus, respectively, and $\kappa \left(x_{i}\right),\lambda \left(x_{i}\right),\mu \left(x_{i}\right)$ the corresponding predicted values from the square function at the *i*th data point. δ corresponds to the nucleus delay and $\nu \left(x_{i}\right)$ to the predicted delay at the *i*th data point. This error metric served as a measure of how well the numerical results match the experimental ones. The response surface optimizer was employed to iteratively adjust the input parameters, optimizing them with respect to all these outputs simultaneously. This way, we aimed to find the optimal set of mechanical properties for every lens that best matched the experimental strain results. Due to the strain-controlled design of the experimental setting, these three derived mechanical properties were independent of the absolute mechanical properties ( $C_{10-N}^{\infty },{\bar{C}}_{10-ANT},{\bar{C}}_{10-POST}$ ). Therefore, in order to approximate the latter, the minimal force measured after the initial relaxation period was taken into account in a second optimization round. Given that the force measurements were affected by substantial experimental noise, force measurements were averaged across each condition, rather than considered individually for each sample.

### 2.6 Statistical analysis

Statistical analysis was conducted with IBM SPSS Statistics (Version 28.0.1.1(14)). Normality of the data was assessed with the Shapiro-Wilk test and accordingly parametric (ANOVA, Student’s t-test) and non-parametric (Independent-Samples Kruskal–Wallis test) tests were applied to compare parameters with normal and skewed distributions, respectively. Bonferroni correction was applied to account for multiple testing. A *p*-value of 0.05 was considered to indicate statistical significance.

## 3 Results

### 3.1 Experimental results

**FIGURE 4:**
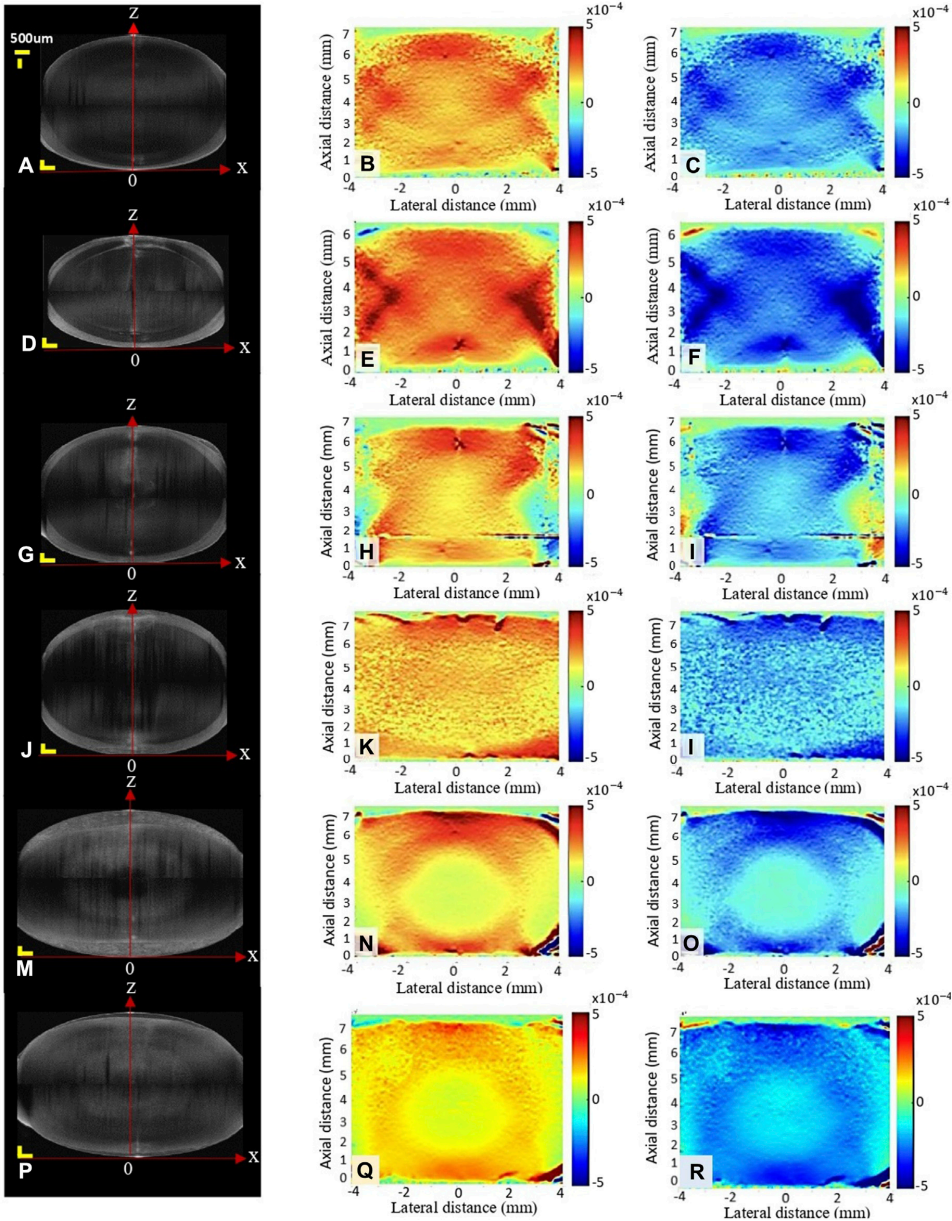
Structural image **(A,D,G,J,M,P)** and axial strain map during relaxation **(B,E,H,K,N,Q)** and compression **(C,F,I,L,O,R)** under different conditions: fresh **(A–C)**, decapsulated **(D–F)**, refrigerator **(G–I)**, MEM **(J–L)**, frozen at −20°C **(M–O)** and frozen at −80°C **(P–R)**. Scale bars in the structural image correspond to 500 μm each.

**FIGURE 5:**
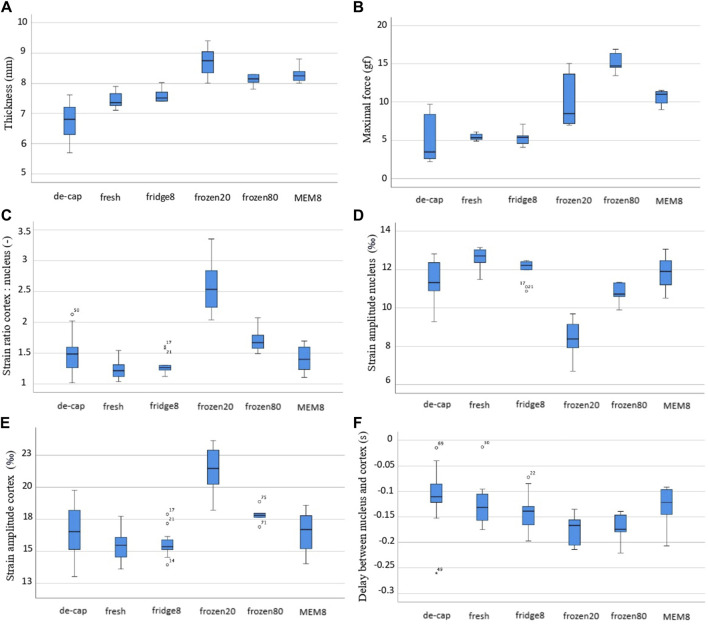
Box and whiskers plot for different experimentally derived parameters: **(A)** lens thickness, **(B)** maximal initial force, **(C)** ratio of cortical to nuclear strain amplitude, **(D)** strain amplitude in the nucleus, **(E)** strain amplitude in the cortex, **(F)** time delay between nucleus and cortex.

The first column of Figure 4 shows the structural scan of the lens of the different groups before compression. As can be noticed, storage in MEM and freezing at both, −20 and −80°C, resulted in a significantly increased lens thickness by +0.85 mm (*p* < 0.001), +1.27 mm (*p* < 0.001) and +0.69 mm (*p* < 0.001), compared to the fresh condition, see also Figure 5A. In contrast, a significant decrease in lens thickness by −0.76 mm (*p* < 0.001) was observed after decapsulation, compared to the fresh group.

**FIGURE 6:**
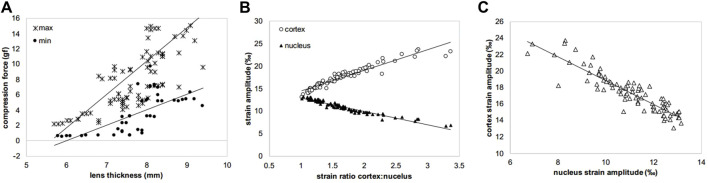
Scatter plots showing the three strongest correlations observed among the experimental variables. **(A)** max and min compression forces versus lens thickness (c_sp_ = 0.775 and c_sp_ = 0.359, *p* < 0.001 and *p* = 0.002), **(B)** strain amplitudes in cortex and nucleus versus strain ratio (c_sp_ = 0.972 and c_sp_ = 0.962, both *p* < 0.001), **(C)** cortical versus nuclear strain amplitudes (c_sp_ = 0.883, *p* < 0.001). c_sp_ means Spearman correlation coefficient.

In agreement with the thickness changes, a higher initial maximal force applied (Figure 5B) in the relaxation test was measured for MEM (11.0 gf, *p* = 0.001) and frozen groups at −20°C and −80°C (8.49 gf, *p* = 0.013 and 9.66 gf, *p* = 0.005), compared to fresh lenses (5.35 gf). The correlation of the maximal force with the lens thickness was statistically significant (r = 0.775, *p* < 0.001). As expected, the minimal force measured at the end of the relaxation was significantly (*p* < 0.001) higher for short relaxation times compared to the longer one (on average 7.52 vs. 2.30 gf). There was also a significant correlation between lens thickness and the min force after relaxation, both at short (c_sp_ = 1.0, *p* < 0.01) and long (c_sp_ = 0.98, *p* < 0.001) relaxation times. Figure 6 presents the strongest correlations observed among the experimental parameters.

The second and third columns in Figure 4 show representative axial strain maps obtained during the oscillating relaxation and compression test. The strain distribution refers to the mean amplitude during relaxation (second column) and compression (third column). Notice that this corresponds to a dynamic strain distribution superposed on top of the pre-strain, which was applied during the initial compression. For better visibility, the pre-strain is omitted here. After freezing at −20°C, the lenses showed the highest strain ratio between the cortex: nucleus with a factor of 2.59 versus the fresh condition with a factor of 1.23 (*p* < 0.001), Figure 5C. Freezing at −80°C induced changes in the same direction but of lower amplitude (1.70, *p* = 0.005). No significant differences were observed between short and long relaxation times. When comparing the absolute strain amplitudes in the nucleus (Figure 5E) and cortex (Figure 5E), freezing at −20°C induced both, a significant decrease in the nuclear strain amplitude by −4.23‰ (*p* < 0.001) and an increase in the cortical strain amplitude by +6.43‰ (*p* < 0.001). Freezing at −80°C induced more subtle changes in nuclear (−1.74‰, *p* < 0.001) and cortical strains (+2.40‰, *p* = 0.005). Decapsulation resulted in a reduced nuclear strain amplitude of −1.30‰ (*p* = 0.010) and an increased cortical strain amplitude by +1.19‰ (*p* = 0.041), compared to the fresh group.

When comparing the different lens conditions in terms of the time delay between nucleus and cortex (Figure 5F), freezing at −20°C and −80°C demonstrated a significantly higher delay than the fresh group (−132 ms vs. −176 ms and −173 ms, *p* ≤ 0.015). No differences were found between short and long relaxation times. Supplementary Figure S1 show the average oscillation pattern in different regions of the lens (cortex and nucleus) for the different conditions.

### 3.2 Numerical results

**FIGURE 7:**
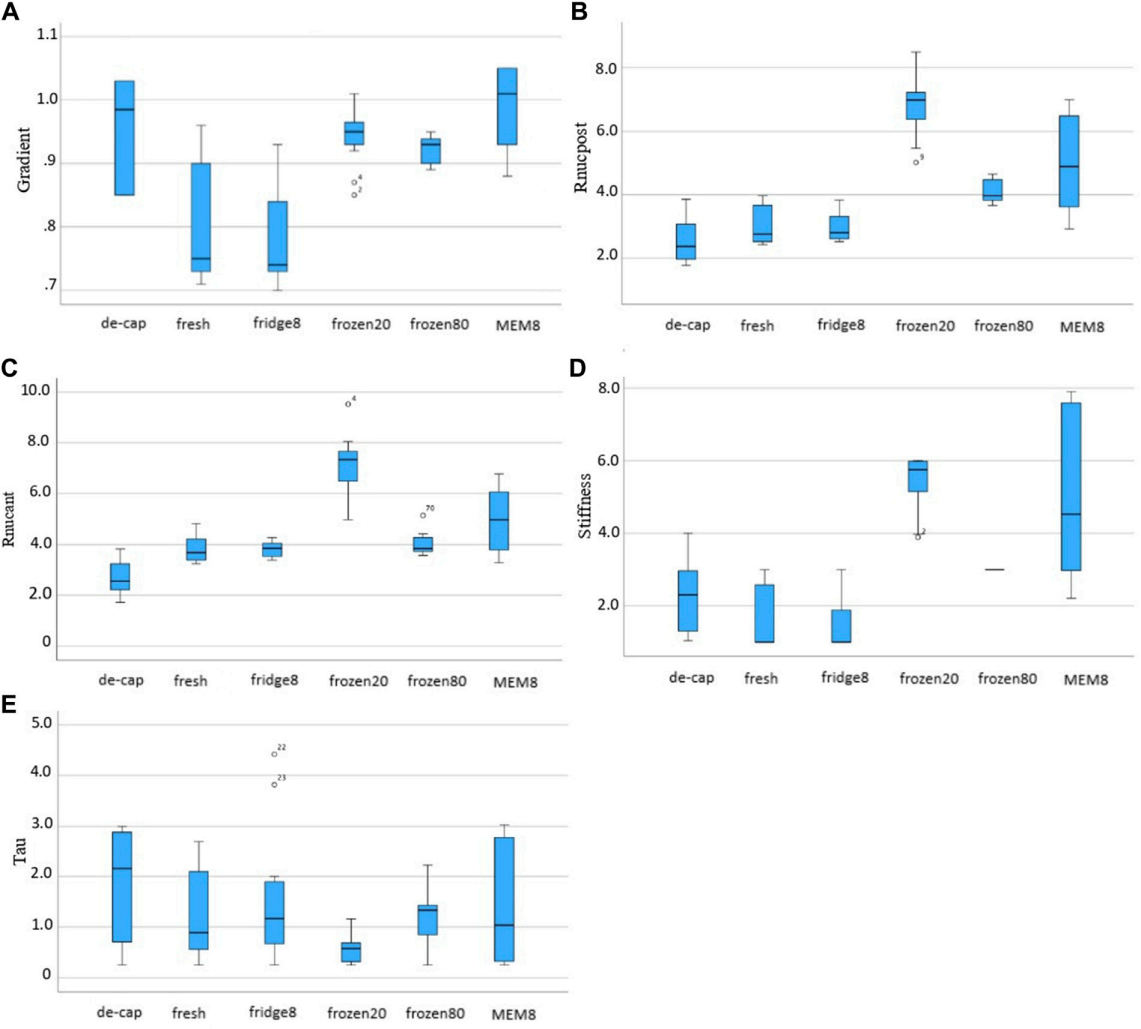
Box and whiskers plots for the inversely derived mechanical parameters: **(A)** form factor F, **(B)** ratio $\frac{C_{10-N}^{\infty }}{{\bar{C}}_{10-POST}}$ , **(C)** ratio $\frac{C_{10-N}^{\infty }}{{\bar{C}}_{10-ANT}}$ , **(D)** stiffness N_stiffness_, **(E)** τ_1_.

Figure 7 presents the mechanical parameters retrieved from iFEA. The inverse fitting was best in the refrigerator condition (91.6%, 91.8%)—with short and long relaxation times, respectively –, followed by MEM (89.8%, 91.6%), decapsulated (88.8%, 86.8%), fresh (87.0%, 82.0%), frozen80 (85.2%, 88.0%) and frozen20 (89.0%, 80.0%) conditions, as determined by the R^2^ (R square) value.

Over all conditions, τ_1_ (1.43 ± 2.07 s vs. 0.82 ± 1.17 s, *p* = 0.023) and the form factor F (0.94 ± 0.16 gf vs. 0.92 ± 0.10 gf, *p* = 0.039) were significantly higher with short compared to long relaxation times. Yet, no significant differences between short and long relaxation times were observed at the level of an individual group.

N_stiffness_ was significantly higher in frozen, both at −20°C and −80°C, and MEM conditions compared to fresh lenses (5.76 ± 0.89, 3.00 ± 0.00 and 4.53 ± 4.67 vs. 1.00 ± 1.69, both *p* < 0.01). Form factor F was significantly higher in decapsulated (0.985 ± 0.18, *p* = 0.005), frozen20 (0.943 ± 0.05, *p* = 0.005) and MEM (1.01 ± 0.13, *p* < 0.001) conditions compared to intact fresh lenses. The ratio between nuclear and cortical strains $\frac{C_{10-N}^{\infty }}{{\bar{C}}_{10-ANT}}$ (7.18 ± 1.17 vs. 3.79 ± 0.51, *p* = .004) and $\frac{C_{10-N}^{\infty }}{{\bar{C}}_{10-POST}}$ (6.78 ± 0.92 vs. 3.04 ± 0.62, *p* < 0.001) were both increased after freezing at −20°C (but not at −80°C), compared to the fresh condition.

## 4 Discussion

Storage of the lens tissue between the time of enucleation and the time when experimental testing is performed is unavoidable when working with human donor eyes. The more important is that an adequate type of storage is chosen that does not alter the lens optical and mechanical properties. While the impact of lens storage was addressed in selected studies^11(p71),12,14^, a spatially-resolved assessment of different interior regions within the lens has not been studied before. Here, we propose OCE in combination with iFEA as a novel tool to assess the internal lens deformations under a controlled compression stimulus for a mechanical characterization at a much higher resolution as before. This study is a further extension of our recent article (Cabeza-Gil et al., 2023), in which we described the combination of compression based OCE with iFEA for the inverse mechanical characterization of fresh *ex vivo* porcine lenses.

The highest impact on the strain distribution within the lens was observed after freezing at −20°C and to a lesser extent after freezing at −80°C. It had been hypothesized earlier that freezing could be associated with structural damage to the lens capsule and this way affect its mechanical behavior. However, despite an increased hydration (thickness) of the lens capsule after freezing was found, its mechanical properties were previously reported to be hardly affected with a 20% decrease in elastic modulus at 0.4 of strain. (Krag and Andreassen, 1998). In contrast, the observations of the current study suggest a substantial change of the internal lens mechanics after freezing, particularly at −20°C. Interestingly, the stiffness in the cortex (weakening) and nucleus (stiffening) experienced changes in opposite directions, which might explain why earlier studies assessing the macroscopic stiffness of the whole lens hardly found a difference. Compared to the preservation in MEM, which experienced a similar increase in lens thickness as the freezing condition, the modified strain distribution across cortex and nucleus appears to be specifically associated with freezing and not related to the lens geometry. The freezing process in porous media such as soil (Talamucci, 2003) has been described to similarly induce a volumetric increase as we observed in the current study, but only if the freezing rate was slow and the overburden pressure was low. We did not observe this effect in the lens. Even though the freezing-induced changes were smaller at −80°C (with a faster freezing rate) compared to −20°C, they were not completely absent. Further increasing the freezing rate to −190°C by immersing the eye globe into liquid nitrogen did burst the eye and thus was not feasible, at least for intact eye globe preservation. The current study is in agreement with earlier findings that suggest storage in the refrigerator (Fisher, 1971) does not significantly affect the lens mechanics.

Overall, our results agree with earlier literature (Ambekar et al., 2020) that the porcine lens has a stiffer nucleus than cortex. (1.98 kPa anterior, 2.93 kPa posterior cortex, 11.9 kPa nucleus). A recent OCE study (Mekonnen et al., 2023) on ARF-stimulated wave propagation concluded that both, the elastic modulus and the shear viscosity coefficient decreased after lens decapsulation (E = 8.14 kPa and η = 0.89 Pa s versus E = 3.10 kPa and η = 0.28 Pa⋅s). Interestingly, in the current study we observed the opposite behavior suggesting an increase in the lens stiffness after decapsulation. This discrepancy might originate from a pre-stress in the lens and capsule due to the presence of the latter, which was not considered in any of these experimental assessments.

The main limitation of the current study is the relatively small sample size and the precision of the load cell to measure force relaxation. Also, it might be perceived that an experimental limitation of the current study was that the maximal forces applied in MEM and frozen groups were higher, because the same lamella distance was used for compression as in the fresh condition. Nevertheless, the fact that no differences were observed in the strain-related measures among short and long relaxation times suggests that the degree of pre-compression had a minor effect on the elastographic assessment. On the other hand, in terms of the inverse FEM, the different degree of pre-strain was adequately considered such that it did not have any negative impact on the numerical results. The primary limitation of the numerical model lies in its own lack of robustness, which is constrained by the potential variability in lens responses under different preservation methods. While the model facilitates the replication of various experimental tests, it necessitates multiple parameters to fully comprehend the crystalline behavior. Advanced material models incorporating features such as porosity and nonlinear viscoelasticity will be explored in forthcoming analyses to potentially yield a more robust depiction of crystalline behavior. In some samples the average (macroscopic strain across the entire lens thickness) differed more than 10% from the theoretically-expected value. Another limitation is that g1 was fixed for all conditions. While this reduced the probability of overfitting, at the same time our methodology was ignorant to any changes in g1 the preservation condition might have caused. Another limitation is that experiments were performed at room temperature instead of body temperature, which reportedly has a measurable effect on the absolute stiffness of the lens (Heilman et al., 2022). However, the primary goal of this study was to assess the impact of different preservation conditions on the lens biomechanics. As all groups were tested at the same temperature and furthermore, we restricted our analyses to relative stiffness ratios, we do not expect any temperature-related bias on the conclusions retrieved in here.

In conclusion, OCT elastography in combination with iFEA is a powerful tool to investigate the mechanical properties of the *ex vivo* lens with high spatial resolution. Our approach was able to detect mechanical changes induced by different preservation techniques, which is critical for a correct interpretation of previous experimental studies on *ex vivo* lenses. At the same time, we could reveal subtle differences in the overall lens’ mechanical behavior due to decapsulation.

## Data Availability

The original contributions presented in the study are included in the article/Supplementary Material, further inquiries can be directed to the corresponding author.
