# Supplementary material for: Storage-induced mechanical changes of porcine lenses assessed with optical coherence elastography and inverse finite element modeling
Source: Front Bioeng Biotechnol. 2024 Jun 13;12:1398659. doi: 10.3389/fbioe.2024.1398659 (PMC11208870; doi:10.3389/fbioe.2024.1398659)
Supplement: Supplementary file 1 [file Image1.pdf]

Supplementary Material

Supplementary Material

1 Supplementary Figures

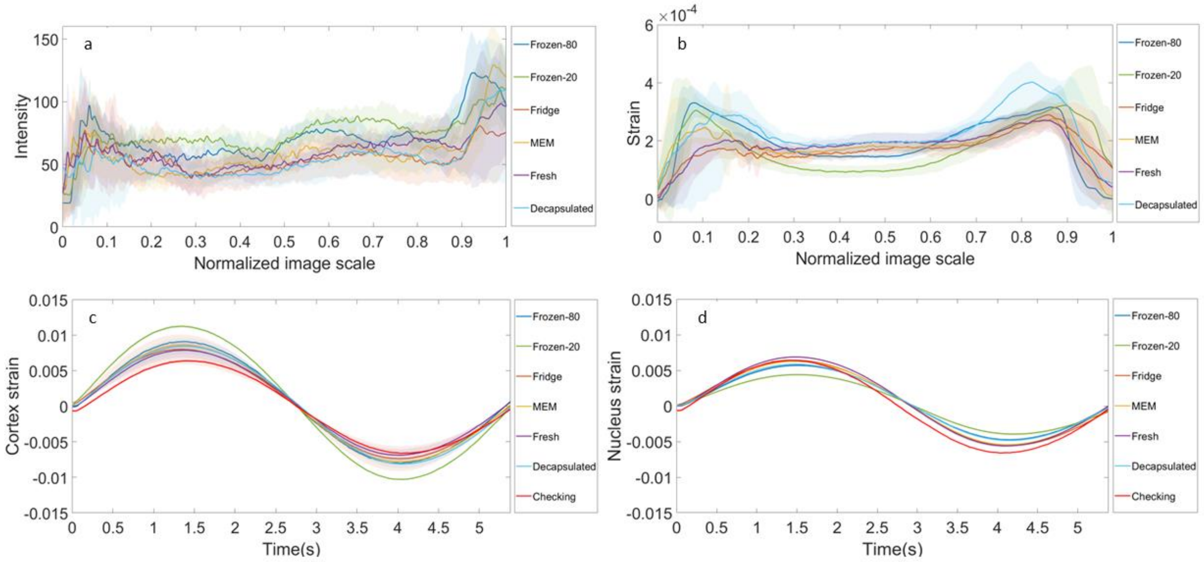

Supplementary Figure 1. (a) Structural reflectivity profile after compression by upper lamella, and (b) axial strain profile for the different conditions. Average oscillation pattern of the axial strain in the (c) cortex and (d) nucleus for different conditions. Error bars represent standard deviation.
